# Crystal structure of *Yersinia pestis* virulence factor YfeA reveals two polyspecific metal-binding sites

**DOI:** 10.1107/S2059798317006349

**Published:** 2017-06-30

**Authors:** Christopher D. Radka, Lawrence J. DeLucas, Landon S. Wilson, Matthew B. Lawrenz, Robert D. Perry, Stephen G. Aller

**Affiliations:** a Graduate Biomedical Sciences Microbiology Theme, University of Alabama at Birmingham, Birmingham, AL 35294, USA; b Office of the Provost, University of Alabama at Birmingham, Birmingham, AL 35294, USA; c Department of Pharmacology and Toxicology, University of Alabama at Birmingham, Birmingham, AL 35294, USA; d Department of Microbiology and Immunology and the Center for Predictive Medicine for Biodefense and Emerging Infectious Diseases, University of Louisville School of Medicine, Louisville, KY 40202, USA; e Department of Microbiology, Immunology, and Molecular Genetics, University of Kentucky, Lexington, KY 40536, USA

**Keywords:** YfeA, *Yersinia pestis*, plague, virulence factor, transition-metal homeostasis, substrate-binding protein (SBP), polyspecificity, X-ray crystallography

## Abstract

Gram-negative bacteria use siderophores, outer membrane receptors, inner membrane transporters and substrate-binding proteins (SBPs) to transport transition metals through the periplasm. The SBPs share a similar protein fold that has undergone significant structural evolution to communicate with a variety of differentially regulated transporters in the cell. In *Yersinia pestis*, the causative agent of plague, YfeA (YPO2439, y1897), an SBP, is important for full virulence during mammalian infection. To better understand the role of YfeA in infection, crystal structures were determined under several environmental conditions with respect to transition-metal levels. Energy-dispersive X-ray spectroscopy and anomalous X-ray scattering data show that YfeA is polyspecific and can alter its substrate specificity. In minimal-media experiments, YfeA crystals grown after iron supplementation showed a threefold increase in iron fluorescence emission over the iron fluorescence emission from YfeA crystals grown from nutrient-rich conditions, and YfeA crystals grown after manganese supplementation during overexpression showed a fivefold increase in manganese fluorescence emission over the manganese fluorescence emission from YfeA crystals grown from nutrient-rich conditions. In all experiments, the YfeA crystals produced the strongest fluorescence emission from zinc and could not be manipulated otherwise. Additionally, this report documents the discovery of a novel surface metal-binding site that prefers to chelate zinc but can also bind manganese. Flexibility across YfeA crystal forms in three loops and a helix near the buried metal-binding site suggest that a structural rearrangement is required for metal loading and unloading.

## Abstract 2

YfeA, a substrate-binding protein that is important for the virulence of *Yersinia pestis*, has two polyspecific metal-binding sites that may play different roles during infection. A flexible lobe at the carboxy-terminus suggests that structural rearrangement is required for metal transfer to binding partners.

## 1. Introduction   

In Gram-negative bacteria, the periplasm plays a crucial role in the homeostasis of essential transition metals by serving as a nutrient bank to supply or deplete the cytoplasm (Andrews *et al.*, 2003 ▸). When resources are limited, extracellular metal ions are sequestered and returned to the cell by high-affinity metal-chelating siderophores and cognate receptors (Andrews *et al.*, 2003 ▸). Trafficking of transition-metal ions through the periplasm is mitigated by siderophores and substrate-binding proteins (SBPs; Couñago *et al.*, 2012 ▸). SBPs similarly chelate and deliver metal ions to inner membrane transporters for passage into the cytoplasm (Argüello *et al.*, 2011 ▸). For pathogenic bacteria, infection presents significant challenges in acquiring nutrients, as metals are in variable abundance according to the site of infection and pathogens must compete with the host for essential metals (Ma *et al.*, 2009 ▸).

**Figure 1:**
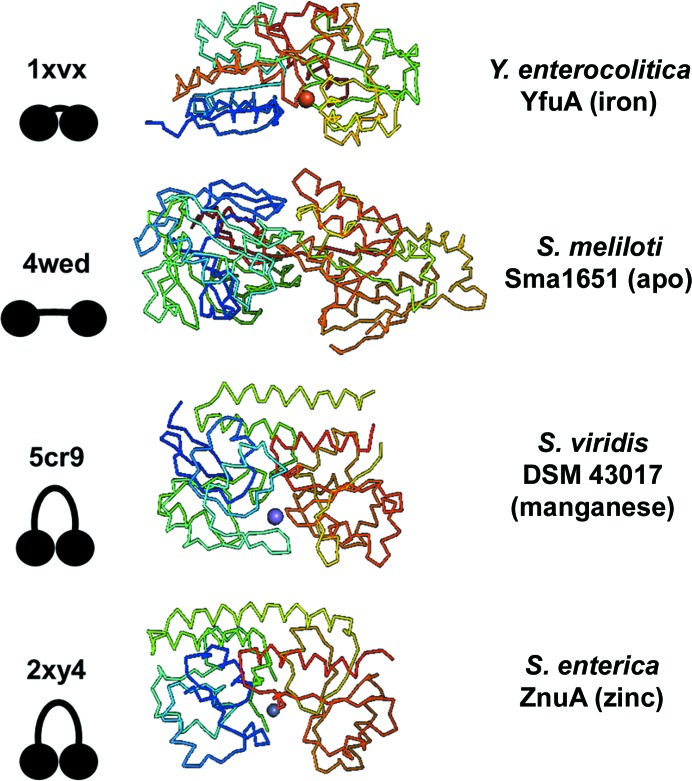
Evolution of the SBP c-clamp. The general structure of an SBP is a c-­clamp. The c-clamp has undergone significant structural evolution. The conformational range that this structure can occupy includes a compact cleft resembling a stone (PDB entry 1xvx; Shouldice *et al.*, 2005 ▸), an elongated cleft resembling a dumbbell (PDB entry 4wed; New York Structural Genomics Research Consortium, unpublished work) and clefts with more curvature that resemble horseshoes (PDB entries 2xy4 and 5cr9; Ilari *et al.*, 2011 ▸; Midwest Center for Structural Genomics, unpublished work). Models for each morphology are included below the respective PDB codes. If a structure is metal-bound, the metal sphere is shown and is colored by CPK chemical convention.

SBPs must have high substrate specificity for the precise regulation of any nutrient and its downstream functions (Lawson *et al.*, 1998 ▸). These polypeptides follow a general c-clamp fold made up of two α/β globular domains that are connected by an α-helical backbone linking region and interdomain hinges composed of β-strands (Couñago *et al.*, 2012 ▸). SBPs also have a canonical metal ion-binding site that is made up of ligands from each domain (Quiocho & Ledvina, 1996 ▸). Metal-binding events may help to stabilize some SBPs by reducing disorder and constraining each globular domain to occupy a single position. Evolution of the c-clamp fold has created diversity in backbone length and SBP size and led to a structural medley of dumbbell and horseshoe morphologies (Fig. 1 ▸). The geometry of the c-clamp is likely to have functional relevance in both the recognition of related inner membrane components as well as metal transport. The SBP structure also determines how deeply buried or exposed a metal-binding site is and may influence metal affinity and exchange. Bacterial systems for nutrient acquisition present intriguing therapeutic targets because transition metals are required for catalysis, replication, metabolism, structure and other functions (Palmer & Skaar, 2016 ▸). Many of these systems are semi-redundant, such that the inhibition of one system is compensated by a related system that transports the same substrate (Plumptre *et al.*, 2014 ▸). Despite this semi-redundancy, some transporters appear to be more critical to cell vitality than others and present stronger phenotypes when inhibited or deleted (Desrosiers *et al.*, 2010 ▸). The Yfe/Sit family of ABC transporters are widespread iron and mangan­ese transporters that play a role in the virulence of a number of pathogens (Bearden & Perry, 1999 ▸; Perry *et al.*, 2012 ▸; Fetherston *et al.*, 2012 ▸). Animal and human serum studies show that *yfeA*, a gene encoding a polyspecific SBP, is among the most upregulated genes in *Yersinia pestis* during bubonic and septicemic plague infection (Rosso *et al.*, 2008 ▸; Perry *et al.*, 2015 ▸). Biochemical analysis indicates that the Yfe system transports manganese and iron, and complements their respective transporters MntH and FeoABC. Double (Δ*yfe* Δ*feo* and Δ*yfe* Δ*mntH*) mutants but not single mutants are significantly attenuated in mouse models of bubonic plague (Bearden & Perry, 1999 ▸; Perry *et al.*, 2007 ▸, 2012 ▸; Fetherston *et al.*, 2012 ▸).

Historically, SBPs were first organized into protein classes that were distinguished by the topology of β-sheets in the globular domains and the connectivity of secondary-structural elements (Fukami-Kobayashi *et al.*, 1999 ▸). Over the last two decades, the number of SBP structures has dramatically increased, prompting a new cluster system to classify SBPs based on both structural and functional properties. In 2010, Berntsson and coworkers established the modern cluster system of SBP classification that contained six distinct clusters (A–F), and in 2016 Scheepers and coworkers updated the cluster system with a new, seventh cluster (G), underscoring the diversity of SBPs that continues to grow through discovery (Berntsson *et al.*, 2010 ▸; Scheepers *et al.*, 2016 ▸). Of the seven clusters of SBPs, clusters A and D contain SBPs involved in transition-metal homeostasis. Cluster A-1 contains SBPs that bind metal ions directly, cluster A-2 contains SBPs that bind chelated metal ions *via* siderophores, and cluster D-4 contains SBPs that bind metal ions directly *via* a synergistic anion (Berntsson *et al.*, 2010 ▸). A key engine to driving SBP structural investigations has been the use of *Escherichia coli* to produce recombinant SBPs from many genera (Lee *et al.*, 1999 ▸; Ilari *et al.*, 2011 ▸; McDevitt *et al.*, 2011 ▸; Gribenko *et al.*, 2013 ▸; Handali *et al.*, 2015 ▸), including *Yersinia pestis* (Shouldice *et al.*, 2005 ▸). *E. coli* has been reliably used to generate recombinant protein that is physiologically relevant and useful for biotechnological applications, namely drug discovery (Lu *et al.*, 2012 ▸; Sanapala *et al.*, 2016 ▸). *E. coli* is particularly useful to study the biology of *Y. pestis* proteins because both *E. coli* and *Y. pestis* are Gram-negative organisms that have similar physiologies and utilize similar cellular machinery. In this work, we report the three-dimensional atomic structure of YfeA, a cluster A-1 SBP, and the discovery of a surface polyspecific metal-binding site that appears to be unique to the *Y. pestis* ortholog, although its function is unknown. We demonstrate by X-ray fluorescence, referred to as energy-dispersive X-ray spectroscopy (EDS), and anomalous X-ray scattering that YfeA specificity at the canonical site is environmentally sensitive. We also present a model for loading and unloading the canonical site based on conformational changes captured from multiple YfeA crystal morphologies.

## 2. Materials and methods   

### 2.1. Cloning, overexpression and purification of YfeA-H_10_   

**Figure 2:**
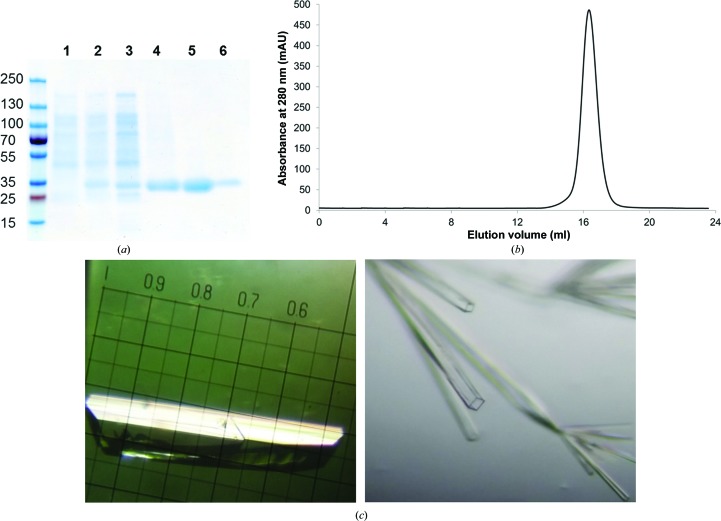
Purification and crystallization of YfeA. (*a*) SDS–PAGE gel showing the enrichment of YfeA during the various steps of purification. Molecular-weight standards are shown on the left (labeled in kDa). Lane 1, uninduced BL21 whole cells. Lane 2, BL21 whole cells induced with IPTG. Lane 3, lysate supernatant fraction following cell disruption with a French press. Lane 4, eluate from nickel-affinity chromatography. Lane 5, eluate from anion-exchange chromatography. Lane 6, gel-filtration peak fraction. (*b*) Superdex 200 10/300 GL gel-filtration chromatogram from YfeA purification. Fractions containing the peak from this chromatogram were concentrated, are represented in lane 6 in (*a*) and were used for crystallography. (*c*) Representative images of YfeA crystals.

The *yfeA* gene (UniProt reference Q56952) was synthesized by and purchased from GenScript (Piscataway, New Jersey, USA) and was inserted into a standard pET-22b vector (Novagen; catalog No. 69744) using NdeI and XhoI cloning sites. In this construct, the vector containing the *yfeA* insertion also codes for a C-terminal His_10_ tag and is expressible in *E. coli*. The plasmid was recovered from ampicillin-resistant *E. coli* colonies and the DNA sequence was verified by the University of Alabama at Birmingham Heflin Center Genomics Core Laboratories. The plasmid was transformed into *E. coli* strain BL21(DE3)pLysS Singles competent cells (Novagen; catalog No. 70236). The transformed cells were grown in LB containing 50 µg ml^−1^ ampicillin with shaking at 225 rev min^−1^ at 37°C. When the OD_600_ reached 0.5–0.6, the temperature was reduced to 16°C and overexpression of YfeA-His_10_ was induced for 16 h with 1 m*M* isopropyl β-d-1-thiogalactopyranoside (IPTG). The same method was used for overexpression in M9 minimal medium (Amresco; catalog No. J863) with a slight modification for induction. Induction in M9 included 1 m*M* IPTG and supplementation with 1 m*M* FeCl_2_, 1 m*M* ZnCl_2_ or 1 µ*M* MnCl_2_. The cells were recovered by centrifugation at 4500*g* for 30 min at 4°C. The cell pellets were resuspended in ice-cold lysis buffer consisting of 20 m*M* Tris pH 7.6, 50 m*M* NaCl, 20 m*M* imidazole, 0.05%(*w*/*v*) NaN_3_ with protease-inhibitor cocktail (cOmplete EDTA-free Protease Inhibitor Cocktail, Roche; catalog No. 05056489001) and were stored at −80°C. After thawing the suspension, the cells were lysed by three cycles in a French press at 10.3 MPa. The crude extract was centrifuged at 48 000*g* for 20 min at 4°C to recover the cell lysate. The lysate supernatant was syringe-filtered using a 0.45 µm membrane unit (Millex; catalog No. SLHV013SL) and subsequently loaded onto a 5 ml HisTrap HP column (GE Healthcare Life Sciences; catalog No. 17-5248-02) that had been pre-equilibrated in lysis buffer. The loaded column was washed with lysis buffer until a stable baseline was achieved and YfeA-His_10_ was eluted with a linear gradient of 0.02–1 *M* imidazole over ten column volumes. Fractions containing YfeA-His_10_ were pooled, buffer-exchanged into ion-exchange buffer consisting of 20 m*M* Tris pH 7.6, 0.05%(*w*/*v*) NaN_3_ and loaded onto a 5 ml HiTrap Q HP column (GE Healthcare Life Sciences; catalog No. 17-1154-01) that had been pre-equilibrated in ion-exchange buffer. The loaded column was washed with ion-exchange buffer until a stable baseline was achieved and YfeA-His_10_ was eluted with a linear gradient of 0–1 *M* NaCl over ten column volumes. Fractions containing YfeA-His_10_ were pooled, concentrated in a stirred cell (Amicon; catalog No. UFSC05001) using a 10 kDa molecular-weight cutoff membrane (Millipore; catalog No. PLGC04310) until the volume reached ∼5 ml and loaded onto a HiLoad 26/600 Superdex 200 pg column (GE Healthcare Life Sciences; catalog No. 28-9893-36) that had been pre-equilibrated in gel-filtration buffer consisting of 20 m*M* bis-tris pH 6.3, 50 m*M* NaCl, 0.05%(*w*/*v*) NaN_3_. Each purification step was monitored by SDS–PAGE (Fig. 2 ▸). The final purified YfeA-His_10_ product in gel-filtration buffer was concentrated in a centrifugal filter unit (Amicon; catalog No. UFC901024) to a final concentration of 18 ± 5 mg ml^−1^ for crystallization. In EDTA experiments, 2 m*M* EDTA was added to the ion-exchange and gel-filtration chromatography buffers, and the purified protein was dialyzed overnight against 100 m*M* EDTA prior to protein concentration and crystallization.

### 2.2. Cloning, overexpression and purification of native YfeA in the context of YfeBCDE   

The previously described pYFE3 plasmid (Bearden *et al.*, 1998 ▸; Bearden & Perry, 1999 ▸) containing the entire YfeA–E locus was transformed into *E. coli* strain BL21-CodonPlus (DE3)-RIPL competent cells (Agilent Technologies; catalog No. 230280). The transformed cells were grown in M9 minimal medium (Amresco; catalog No. J863) containing 50 µg ml^−1^ ampicillin with shaking at 225 rev min^−1^ at 37°C for 9 h. The cells were recovered by centrifugation at 4500*g* for 30 min at 4°C. The cell pellets were resuspended in ice-cold lysis buffer consisting of 20 m*M* phosphate buffer pH 7.6, 50 m*M* NaCl and were stored at −80°C. After thawing the suspension, the cells were centrifuged at 4000*g* for 20 min at 4°C and then resuspended in 0.2 *M* Tris pH 8.0, 0.4 *M* NaCl, 2 m*M* EDTA to begin cell fractionation. The cells were incubated over ice for 20 min with occasional inversion to counter sedimentation. The cells were then re-centrifuged at 4000*g* for 20 min at 4°C, resuspended in 10 m*M* Tris pH 8.0 and incubated over ice for an additional 20 min with occasional inversion to counter sedimentation. Spheroplasts were pelleted by centrifugation at 48 000*g* for 20 min at 4°C and the periplasmic fraction was recovered from the supernatant. The spheroplasts were resuspended in 20 m*M* phosphate buffer pH 7.6, 50 m*M* NaCl and lysed by three cycles in a French press at 10.3 MPa. The crude extract was centrifuged at 48 000*g* for 20 min at 4°C to recover the cell-lysate supernatant. The membranes were separated from the lysate supernatant by centrifugation at 70 000*g* for 45 min at 4°C and the cytoplasmic fraction was recovered from the supernatant. The pellet was then resuspended in 4%(*v*/*v*) Triton X-100 and mixed by gentle rocking overnight at 4°C. The mixture was then centrifuged at 70 000*g* for 45 min at 4°C, and the inner membrane fraction was recovered from the supernatant. The pellet containing the outer membrane fraction was then resuspended in 0.1%(*w*/*v*) DDM. Immediately after extraction, the periplasmic fraction was syringe-filtered using a 0.45 µm membrane unit (Millex; catalog No. SLHV013SL) and subsequently loaded onto a 5 ml HiTrap Q HP column (GE Healthcare Life Sciences; catalog No. 17-1154-01) that had been pre-equilibrated in ion-exchange buffer. The loaded column was washed with ion-exchange buffer until a stable baseline was achieved and native YfeA was eluted with a linear gradient of 0–1 *M* NaCl over ten column volumes. The remainder of the purification from this point was identical to that for YfeA-H_10_.

### 2.3. Mass-spectrometry data collection   

The stained bands were excised and the stain was removed by an overnight wash in 50% 100 m*M* ammonium bicarbonate/50% acetonitrile. Disulfide bonds were reduced using 25 m*M* dithiothreitol at 50°C for 30 min followed by alkylation of the free thiol groups with 55 m*M* iodoacetamide for 30 min in the dark. After the removal of excess alkylating agent, the gel pieces were evaporated to dryness prior to reswelling in 100 m*M* ammonium bicarbonate buffer and overnight digestion using mass-spectrometry-grade trypsin (12.5 ng ml^−1^). Tryptic peptides were extracted using a solution of 1% formic acid in water and acetonitrile (50:50) and then evaporated to dryness in a Speedvac. The samples were resuspended in 30 µl double-distilled water (ddH_2_O) with 0.1% formic acid for mass-spectrometric evaluation.

An aliquot (5 µl) of each digest was loaded onto a Nano cHiPLC (200 µm × 0.5 mm, ChromXP C18-CL, 3 µm, 120 Å) reverse-phase trap cartridge (Eksigent, Dublin, California, USA) at 2 µl min^−1^ using an Eksigent 415 LC system autosampler. After washing the cartridge for 10 min with 0.1% formic acid in ddH_2_O, the bound peptides were flushed onto a Nano cHiPLC column (200 µm internal diameter × 15 cm, ChromXP-C18-CL, particle size 3 µm, pore size 120 Å; Eksigent, Dublin, California, USA) with a 15 min linear (5–50%) acetonitrile gradient in 0.1% formic acid at 1000 nl min^−1^ using an Eksigent Nano1D+LC (Dublin, California, USA). The column was washed with 95% aceto­nitrile/0.1% formic acid for 5 min and then re-equilibrated with 5% acetonitrile/0.1% formic acid for 5 min. A SCIEX 5600 Triple-TOF mass spectrometer (Sciex, Toronto, Canada) was used to analyze the protein digest. The ion-spray voltage was 2300 V and the declustering potential was 80 V. The ion-spray and curtain gases were set at 69 and 172 kPa, respectively. The interface heater temperature was 120°C. The eluted peptides were subjected to a time-of-flight survey scan from *m*/*z* 400 to 1250 to determine the top 20 most intense ions for MS/MS analysis. Product-ion time-of-flight scans at 50 ms were carried out to obtain the tandem mass spectra of the selected parent ions over the *m*/*z* range 400–1500. Spectra were centroided and de-isotoped by the *Analyst* software v.1.6 TF (Sciex). A β-galactosidase trypsin digest was used to establish and confirm the mass accuracy of the mass spectrometer.

The tandem mass-spectrometry data were processed to provide protein identification in-house using the *Protein Pilot* 4.5 search engine (Sciex) using the *Y. pestis* UniProt protein database and using a trypsin-digestion parameter. Proteins of significance were accepted on the criteria of having at least two peptides detected with a confidence score of 95% or greater.

### 2.4. YfeA crystallization   

Crystallization conditions were determined by a rational approach comparing the crystallization conditions of YfeA orthologs in the Protein Data Bank (PDB). YfeA-His_10_ and native YfeA protein crystals were grown by the hanging-drop and sitting-drop vapor-diffusion methods at 293 K in gel-filtration buffer with 30–40%(*w*/*v*) PEG 4000. The final, optimized condition that led to the highest resolution data set was 20 m*M* bis-tris pH 6.3, 50 m*M* NaCl, 0.05%(*w*/*v*) NaN_3_, 30%(*w*/*v*) PEG 4000. Drops consisted of YfeA-His_10_ or native YfeA plus reservoir solution in 1:1, 1:2 and 2:1 ratios for hanging-drop setup. Crystals grew in two main morphologies, tetragonal prisms and thin plates, and were directly flash-cooled in liquid nitrogen. Co-crystallization experiments included the addition of 10 m*M* ZnCl_2_, 10 m*M* MnCl_2_ or 10 m*M* FeCl_2_ and 1 m*M* β-mercaptoethanol to the crystallization-drop solution. The final, optimized condition that produced the crystals used for anomalous data collection and led to the confirmation of site 2 was 20 m*M* bis-tris pH 6.3, 50 m*M* NaCl, 0.05%(*w*/*v*) NaN_3_, 32%(*w*/*v*) PEG 4000. In the EDTA experiments, purified YfeA-His_10_ protein was co-incubated with 2 m*M* EDTA in 20 m*M* bis-tris pH 6.3, 50 m*M* NaCl, 0.05%(*w*/*v*) NaN_3_, 35%(*w*/*v*) PEG 4000. Soaking experiments with YfeA-H_10_ included 3 and 4 h soaks in 10 m*M* ZnCl_2_, 10 m*M* MnCl_2_ or 10 m*M* FeCl_2_ + 1 m*M* β-mercaptoethanol. Some crystals changed from clear to yellow during soaking.

### 2.5. X-ray data collection, structure solution and refinement   

Diffraction data were collected at 100 K on the Southeast Regional Collaborative Access Team (SER-CAT) 22-ID beamline and the General Medical Sciences and Cancer Institutes Structural Biology Facility (GM/CA) 23-ID-B and 23-ID-D beamlines at the Advanced Photon Source (APS), Argonne National Laboratory, and the Canadian Macromolecular Crystallography Facility (CMCF) 08ID-1 beamline at the Canadian Light Source (CLS), University of Saskatchewan. The anomalous X-ray scattering *K*-edge energies for Zn, Mn and Fe were empirically determined for each data collection. The data-collection strategy for each crystal was determined using the *iMosflm* Strategy function (Battye *et al.*, 2011 ▸), targeting ≥95% completeness for anomalous data. Energy-dispersive X-ray spectroscopy fluorescence data were collected at 100 K at the General Medical Sciences and Cancer Institutes Structural Biology Facility (GM/CA) 23-ID-B and 23-ID-D beamlines at the Advanced Photon Source, Argonne National Laboratory. The data were merged and scaled using *HKL*-2000 (Otwinowski & Minor, 1997 ▸). The data completeness and *R*_merge_ were used to determine the resolution limit. Phases were determined by SAD using *AutoSol* as implemented in the *PHENIX* suite (Adams *et al.*, 2010 ▸). Model building and refinement were performed using *AutoBuild* and *PHENIX*. In the case of building models of YfeA molecules with Zn atoms bound to ancillary surface sites, the grouping of ancillary sites that did not co-load with site 2 contained two Zn atoms that shifted slightly out of anomalous density during refinement. Occupancy and/or anomalous refinement did not improve their final locations, so we set the occupancies of these atoms to 1.00 and manually moved them back into anomalous density for the final PDB file. The figures were generated using *PyMOL* (http://www.pymol.org).

### 2.6. Sequence alignment   

YfeA orthologs were identified by searching the YfeA primary structure against the PDB (Berman *et al.*, 2000 ▸). Sequence alignment of YfeA with orthologs was performed using *Clustal Omega* at http://www.ebi.ac.uk/Tools/msa/clustalo/.

## 3. Results   

### 3.1. YfeA utilizes a c-clamp architecture   

**Figure 3:**
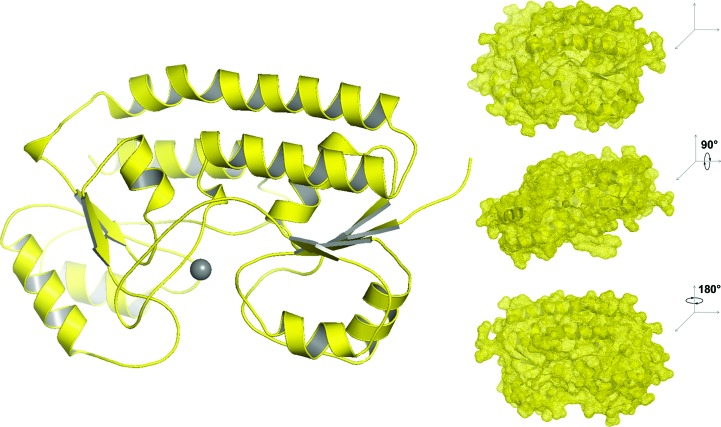
Overall structure of YfeA. YfeA is a c-clamp. The structure contains two globular lobe domains that are interconnected by an α-helical backbone. The position of the site 1-bound metal is shown. Surface rendering shows that site 1 is deeply buried in the c-clamp.

**Figure 4:**
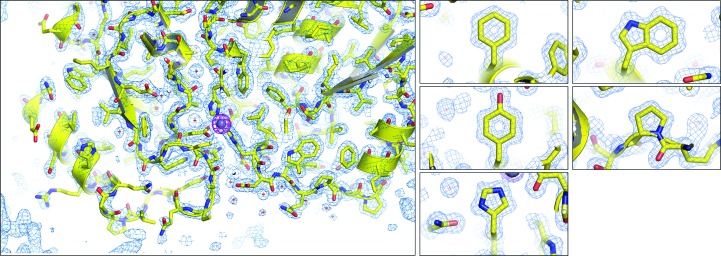
YfeA model fit. Model overlay of anomalous electron density contoured at 5σ (magenta mesh) with 2*F*_o_ − *F*_c_ electron density (blue mesh) at site 1. The enlarged images at a proline and aromatic residues show the map quality, highlighting apparent holes in ringed structures.

YfeA (YfeA-H_10_) was isolated to apparent purity (>99%) and homogeneity by nickel-affinity, anion-exchange and gel-filtration chromatography (Fig. 2 ▸ *b*). YfeA migrates as a single 30 kDa band on an SDS–PAGE gel (Fig. 2 ▸ *a*). Diffraction-quality crystals were grown by the optimization of initial hits that were identified by rational screening (Fig. 2 ▸ *c*), and the Bravais lattice belonged to the orthorhombic crystal system *P*2_1_2_1_2_1_. By varying the protein-expression conditions, we observed crystallization of YfeA in three crystal forms. In this report, the lattices with edges 42 × 52 × 113, 62 × 66 × 67 and 55 × 67 × 82 Å will be referred to as crystal forms 1, 2 and 3, respectively. The structure of crystal form 1 was solved by single anomalous dispersion (SAD), and the structures in the other crystal forms were determined by molecular replacement (MR). 80% of all residues in crystal form 1 were built using *AutoBuild* in *PHENIX*, and the generated model was refined to an *R*_work_ and *R*_free_ of 17.88 and 19.03%, respectively, and used for MR and refinement of crystal form 2 and crystal form 3 (Adams *et al.*, 2010 ▸). The high-quality diffraction data enabled the resolution of holes in aromatic and proline residues (Fig. 3 ▸). Strong anomalous data collected at the Zn *K*-shell electron energy absorption edge (*K* edge) clearly identified the canonical site in YfeA (Fig. 4 ▸). Data statistics are provided in Tables 1 ▸ and 2 ▸.

**Figure 5:**
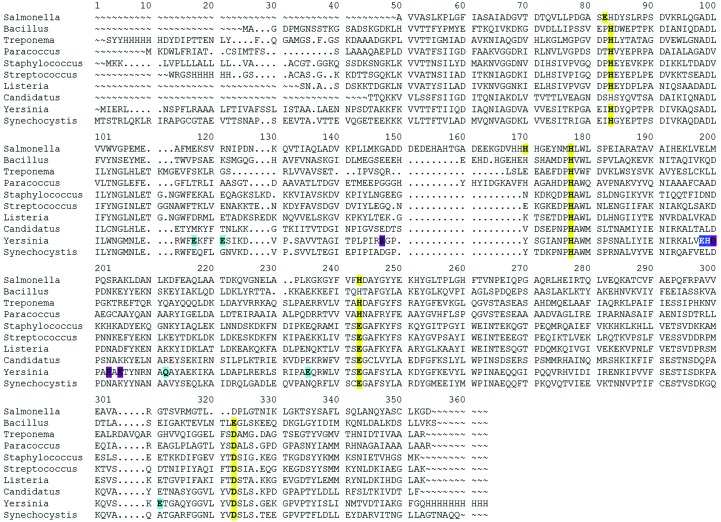
Primary-structure alignment of YfeA ortholog SBPs. YfeA, designated as *Yersinia*, aligned with orthologs from other genera, including Gram-positive genera, with known three-dimensional structures: *Salmonella enterica* zinc-binding protein ZnuA (PDB entry 2xy4; Ilari *et al.*, 2014 ▸), *Bacillus subtilis* manganese-binding protein YcdH (PDB entry 2o1e; Northeast Structural Genomics Consortium, unpublished work), *Treponema pallidum* zinc-binding protein TroA (PDB entry 1toa; Lee *et al.*, 1999 ▸), *Paracoccus denitrificans* zinc-binding protein Pden1597 (PDB entry 4xrv; Handali *et al.*, 2015 ▸), *Staphylococcus aureus* manganese-binding protein MntC (PDB entry 4k3v; Gribenko *et al.*, 2013 ▸), *Streptococcus pneumoniae* manganese-binding protein PsaA (PDB entry 3ztt; McDevitt *et al.*, 2011 ▸), *Listeria monocytogenes* manganese-binding protein MntA (PDB entry 5i4k; Center for Structural Genomics of Infectious Diseases, unpublished work), *Candidatus* Liberibacter asiaticus manganese-binding protein CLas-ZnuA2 (PDB entry 4cl2; Sharma *et al.*, 2015 ▸) and *Synechocystis* sp. PCC 6803 manganese-binding protein MntC (PDB entry 1xvl; Rukhman *et al.*, 2005 ▸). Site 1 ligands from all orthologs are shown in yellow. Site 2 in YfeA is shown in dark blue. Tertiary, ancillary sites that co-load with site 2 are shown in purple, and sites that do not co-load with site 2 are shown in light blue.

In all crystal forms, YfeA is a c-clamp with amino-terminal and carboxy-terminal α/β globular domains that are connected by an α-helical backbone linking region (Fig. 3 ▸). Each α/β domain–backbone interface is distinguishable by β-strand hinges that are similar to those in other SBPs. At the arch of the c-clamp is a tetracoordinate canonical site composed of His76, His141, Glu207 and Asp282. These residues are in alignment agreement with other SBPs of known structure from Gram-positive and Gram-negative genera (Fig. 5 ▸). This canonical metal-binding site is the primary metal-binding site in YfeA and we refer to it as site 1.

### 3.2. YfeA is a polyspecific SBP and site 1 metal occupancy depends on environmental conditions   

**Figure 6:**
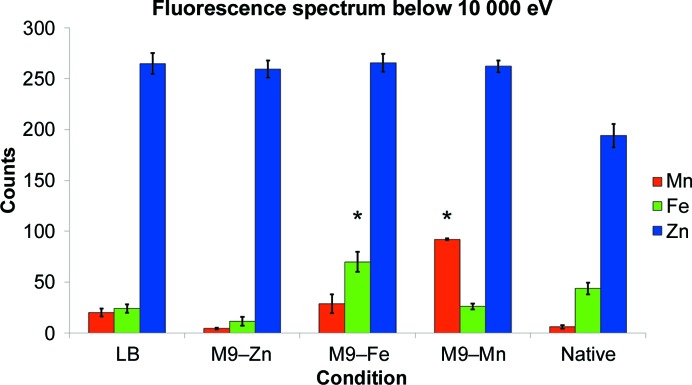
EDS spectra of YfeA crystals. The EDS data show changes in the metal signal profiles according to cell-growth conditions. Data are represented as the mean ± 1σ. Asterisks indicate changes that are statistically significant. In M9 iron experiments, the change in iron had a calculated *p* value of 2.6 × 10^−4^ with α = 0.01. In M9 manganese experiments, the change in manganese had a calculated *p* value of 7.9 × 10^−6^ with α = 0.01. Zinc remains the dominant substrate in native, untagged YfeA that is overexpressed in the context of YfeBCDE and purified from the periplasm.

**Figure 7:**
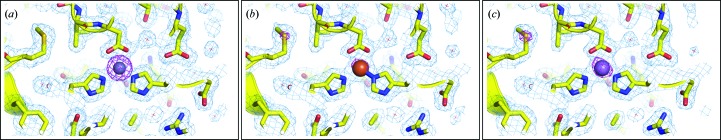
Anomalous electron density at site 1. Enlarged images of the structural model at site 1 with an overlay of anomalous electron density (magenta mesh) with 2*F*_o_ − *F*_c_ electron density (blue mesh). In all maps, the anomalous density is contoured at 5σ to emphasize the differences in signal for manganese (*a*), iron (*b*) and zinc (*c*). In (*a*) and (*b*) there is additional anomalous density from a methionine S atom that does not appear for the same S atom in the zinc data. These images were collected using data from the M9–Zn experiment.

The EDS spectra of YfeA crystals from protein purified from *E. coli* cells grown in Luria–Bertani broth (LB) revealed strong florescence emission signals from zinc and weak signals from iron and manganese (Fig. 6 ▸). Although this observation suggests that the primary ligand at site 1 is zinc, further inspection was needed as nutrient-rich conditions contain unequal abundances of free metals in solution, and zinc is the most abundant transition metal in LB (Outten & O’Halloran, 2001 ▸). To better probe the site 1 metal preference, YfeA protein was purified from *E. coli* cells grown in M9 minimal medium (M9) that was supplemented with either zinc, manganese or iron in separate experiments during YfeA overexpression. Minimal medium was used in many of the experiments in this study because minimal medium constrains cells to maintain tighter regulation over cellular processes compared with cells growing in LB, thereby enabling the observation of relevant cellular processes that may otherwise be masked by cellular physiology. By design, metal supplementation ensured that whichever metal was added became the most abundant metal in solution and was available for YfeA chelation. Surprisingly, under these conditions the strongest fluorescence emission from YfeA crystals was still from zinc, although as expected the greatest disparity between the zinc signal and the iron or manganese signal occurred from M9 with zinc supplementation (Fig. 6 ▸). M9 iron supplementation caused a threefold increase in iron emission relative to the iron signal from nutrient-rich conditions (Fig. 6 ▸). M9 manganese supplementation caused a fivefold increase in manganese emission relative to the manganese signal from nutrient-rich conditions (Fig. 6 ▸). 1 µ*M* MnCl_2_ was used for manganese supplementation instead of the 1 m*M* metal concentration that was used in the M9 zinc and iron experiments because higher levels of manganese inhibited cell growth and reduced the biomass prior to protein purification. 1 µ*M* MnCl_2_ was determined to be an acceptable compromise between final biomass and metal supplementation. Granted, 1 µ*M* MnCl_2_ is still tenfold greater than the manganese level in LB (Outten & O’Halloran, 2001 ▸). ANOVA statistical testing indicates that the changes in manganese and iron emission signals from their respective M9 experiments are statistically relevant, with the iron change yielding a *p* value of 2.6 × 10^−4^ at α = 0.01 and the manganese change yielding a *p* value of 7.9 × 10^−6^ at α = 0.01. Although zinc emission always produced the strongest signal of the metals, it is evident from EDS that changes in the growth conditions elicit changes in the relative abundance of metals in YfeA. To show that these changes correlate with metal incorporation at site 1, anomalous X-ray scattering data were collected at the zinc, iron and manganese *K* edges from crystals grown from M9 experiments (Fig. 7 ▸). By collecting X-ray data at the various *K* edges, the anomalous contribution from each metal provided another quantitative tool to precisely evaluate the ligand bound to site 1. Additionally, the structural data confirmed that the only ordered metal that could contribute anomalous signal was bound to site 1, and given that YfeA substrates were absent from purification buffers and crystallization conditions, the metal bound to site 1 must have been sequestered by YfeA *in vivo*. In the anomalous X-ray data, the strongest signal at site 1 is from zinc (Fig. 7 ▸ *a*), but there is positive signal for iron (Fig. 7 ▸ *b*) and manganese (Fig. 7 ▸ *c*). The anomalous signals for each metal increased during their respective M9 experiments and are in good agreement with the EDS data. As an internal control for energy changes to longer wavelengths (∼1.28 Å for zinc, ∼1.74 Å for iron and ∼1.89 Å for manganese), an S atom from Met144 is visible, contributing a positive weak anomalous signal, shown as pink mesh, that intensified as the experimental wavelength approached the anomalous sulfur edge (Figs. 7 ▸ *a*, 7 ▸ *b* and 7 ▸ *c*). Elsewhere in the model, an additional S atom from Met99 was also observed to contribute a positive weak anomalous signal at longer wavelengths. Across all conditions and crystals tested in this study, fluorescence experiments were only positive for zinc, manganese and iron, and were negative for all other metals, including divalent species. Taken together, the EDS and anomalous X-ray scattering data show that site 1 is predominantly loaded with zinc but can incorporate manganese or iron under nutrient-limiting conditions *in vivo*. The inter­atomic distances of site 1 ligands are 3.0 Å ≤ *x*–*x*1 ≤ 3.6 Å. In proteins, the mean interatomic distances from ligand to zinc, iron and manganese are 2.27, 2.64 and 2.23 Å, respectively (Goyal & Mande, 2008 ▸). Additionally, zinc, iron and manganese can bind with tetracoordinate geometry (Lide, 2001 ▸). Based on this analysis, the interatomic distances of site 1 ligands can integrate zinc, iron and manganese, further supporting the hypothesis that YfeA site 1 is polyspecific. Control EDS experiments with YfeA protein exposed to EDTA, in which any metals bound to the His_10_ tag would be removed, determined that nonspecific metal binding to the tag cannot have influenced the EDS signatures of zinc, manganese or iron binding.

### 3.3. Without YfeBCDE, YfeA site 1 is always occupied in *E. coli* and cannot be stripped by EDTA   

YfeA homologs have previously been produced in the apo state by dialyzing purified protein against EDTA prior to crystallization (Yatsunyk *et al.*, 2008 ▸; Abate *et al.*, 2014 ▸; Sharma *et al.*, 2015 ▸). EDTA was similarly used in this work to attempt to generate apo YfeA protein. Initial dialysis against 10 m*M* EDTA did not remove the metal from site 1; therefore, 2 m*M* EDTA was added to the anion-exchange and gel-filtration buffers and 100 m*M* EDTA was used for overnight dialysis. The X-ray data showed that site 1 was still occupied after this treatment; hence, we sought co-crystallization with 1 m*M* EDTA. EDTA co-crystallization also did not strip the metal from site 1; however, these studies uncovered crystal form 3 and indicated that site 1 is deeply buried (Fig. 3 ▸). For YfeA to surrender its metal, a significant conformational change, unfolding, proteolytic or other enzymatic event appears to be essential. The observation that site 1 is always metal-bound suggests that either YfeA spontaneously scavenges metal or, albeit unlikely, requires a binding partner for metal loading that is also present in *E. coli*. Although site 1 is always metal-bound in our studies, we do not exclude the possibility of an *E. coli* mechanism that may unload site 1 *in vivo*.

### 3.4. YfeA has a secondary polyspecific metal-binding site   

**Figure 8:**
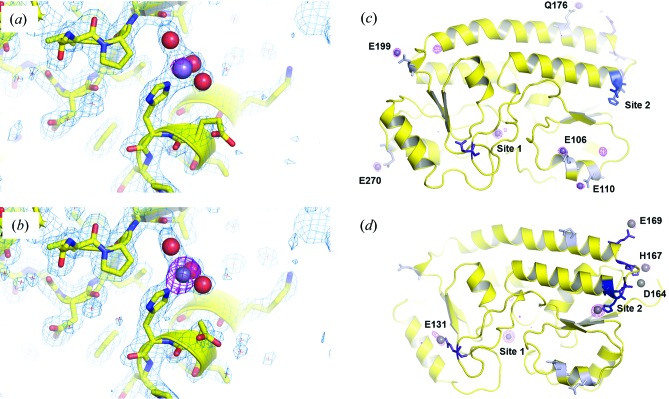
Polyspecificity at site 2 and additional metal-binding sites in YfeA. (*a*, *b*) Enlarged images of the structural model at site 2 with an overlay of anomalous electron density (magenta mesh) with 2*F*_o_ − *F*_c_ electron density (blue mesh). In both maps, the anomalous density is contoured at 5σ to show the differences in signal for manganese (*a*) and zinc (*b*). Water molecules are shown as red spheres, and manganese and zinc ions are shown as spheres colored by CPK chemical convention. (*c*, *d*) Cartoon representation of all metal-binding sites in YfeA with an overlay of anomalous electron density (magenta mesh) contoured at 5σ. (*c*) Ancillary sites that do no co-load with site 2. (*d*) Ancillary sites that co-load with site 2.

Prior to realising that site 1 is always occupied, YfeA co-crystallization experiments with zinc, iron or manganese were conducted to attempt to influence metal incorporation into site 1. Although co-crystallization did not have any impact on site 1, the experimental outcomes were unique to each metal. Co-crystallization experiments with iron and β-mercapto­ethanol [used to maintain iron(II) oxidation in the crystallization drop] resulted in clear drops that never produced crystals, while zinc co-crystallization experiments resulted in immediate heavy precipitation. Manganese co-crystallization experiments produced diffraction-quality crystals which, after anomalous X-ray data processing, revealed a secondary metal-binding site referred to in this manuscript as site 2. After further investigation, site 2 was determined to be polyspecific and capable of binding manganese and zinc. In an experimental set of >40 YfeA crystals, site 2 was never observed to be metal-bound unless manganese was included in the co-crystallization experiment but, curiously, zinc is the preferred metal for site 2. This observation was initially peculiar, as it suggested that site 2 was highly specific for zinc and was able to bind contaminating zinc ions from the MnCl_2_ reagent while also binding manganese. Furthermore, the anomalous signal was always stronger from zinc than manganese at site 2, and could not be manipulated otherwise. Site 2 is a surface site made up of the ligands Glu162 and His163, with ordered water molecules filling the vacant orbitals of bound metal ions (Figs. 8 ▸ *a* and 8 ▸ *b*). Soaking YfeA crystals in manganese or zinc could not load site 2, and multiple trials of consecutive back-soaking experiments in crystallization buffer void of mang­anese or zinc could not remove metal from site 2. Co-crystallization and back-soaking experiments indicate that the loading of site 2 is only observable through crystallization, which irreversibly traps metal bound to site 2 and precludes exogenous metal from binding in site 2. Soaking experiments indicate that site 2 ligands may require conformational flexibility for metal loading, which is only available while YfeA is still in a monomeric form in solution prior to the impasse imposed by crystallization.

Should site 2 be loaded *in vivo*, we expect the bound metal to be readily exchangeable and to play a role in communication with the Yfe transporter and/or direct metal transport. To provide an additional line of evidence that zinc was bound at site 2, albeit as a MnCl_2_ chemical contaminant, a zinc–manganese co-crystallization titration experiment was performed to determine a zinc concentration that would not precipitate YfeA and still produce crystals, as well as increase the zinc occupancy at site 2. The maximum concentration of zinc that produced crystals with metal bound to site 2 was 10 µ*M* ZnCl_2_/1 m*M* MnCl_2_. Co-titration increased the difference between the amount of zinc loaded at site 2 relative to manganese, making zinc, which was already favored at site 2 in the original experiment, even more represented than before. The co-titration experiment also suggests that mangan­ese influences the YfeA structure in a way that cannot be detected crystallographically. A zinc titration was performed in the absence of manganese at a lower concentration range to avoid YfeA precipitation, but the crystals that grew did not have any metal bound to site 2. All structures with a metal-bound site 2 belonged to crystal form 2.

### 3.5. YfeA can freely bind zinc from bulk solvent at low-affinity ancillary metal-binding sites   

Crystal-soaking experiments were performed using manganese, iron with β-mercaptoethanol, and zinc. Since YfeA contains no disulfide bonds, reducing agents do not perturb its structure. Similarly to co-crystallization experiments, only one type of metal in the soaking experiments showed metal binding to YfeA. Soaks in zinc yielded crystals with surface ancillary metal-bound sites; however, back-soaking experiments in crystallization buffer void of zinc were able to remove the metal from all of the ancillary sites, suggesting that these sites are exchangeable. From these experiments, a total of nine sites were discovered: Glu106, Glu110, Glu131, Asp164, His167, Glu169, Gln176, Glu199 and Glu270 (Figs. 8 ▸ *c* and 8 ▸ *d*). Given that these sites can only be loaded through soaking, co-crystallization experiments with zinc may have immediately precipitated YfeA because the additional Zn atoms that bind each YfeA molecule may have had a deleterious effect on the electrostatics that would have otherwise stabilized crystal packing. Metal loading at ancillary sites varies according to state of site 2. When site 2 is apo, Glu106, Glu110, Gln176, Glu199 and Glu270 can be loaded (Fig. 8 ▸ *c*), whereas when site 2 is metal-bound Glu131, Asp164, His167 and Glu169 can be loaded (Fig. 8 ▸ *d*). It is interesting that the ancillary sites that can be co-loaded with site 2 are predominantly in the immediate proximity of site 2, whereas the ancillary sites that do not co-load with site 2 are distant from site 2 (Figs. 8 ▸ *c* and 8 ▸ *d*).

### 3.6. YfeA in the context of the Yfe transporter is predominantly zinc-bound   

**Figure 9:**
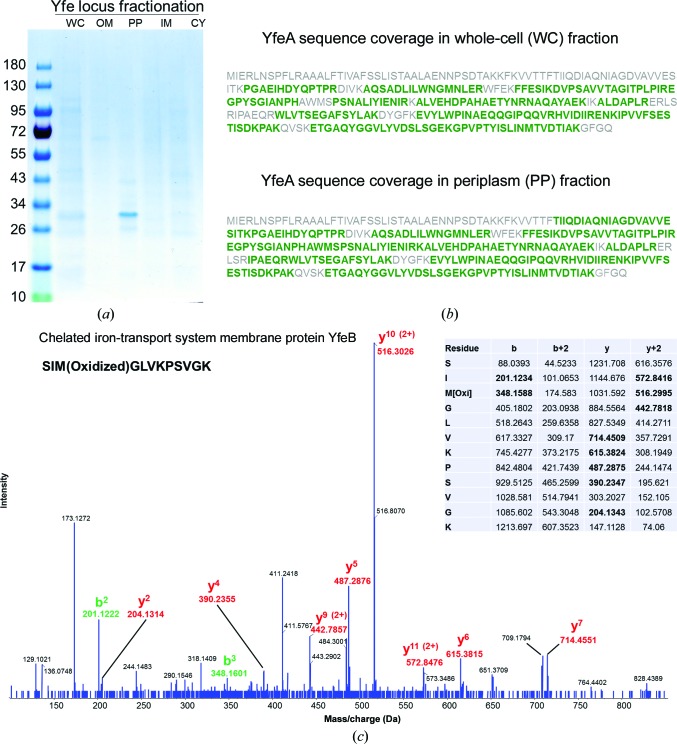
Fractionation of *E. coli* cells overexpressing the Yfe locus by an endogenous promoter. (*a*) SDS–PAGE gel showing expression of Yfe locus components and localization of YfeA in the *E. coli* BL21-CodonPlus (DE3)-RIPL periplasm. Molecular-weight standards are shown on the left (labeled in kDa). Lane WC, whole cells. Lane OM, outer membrane fraction. Lane PP, periplasm fraction. Lane IM, inner membrane fraction. Lane CY, cytoplasm fraction. (*b*) Mass-spectrometric sequence coverage of YfeA, shown in green, in the whole-cell and periplasm fractions. (*c*) Mass spectrum of YfeB in the whole-cell fraction.

To explore the structure of metal binding of YfeA in protein co-expressed with its downstream physiological partners, YfeBCDE, wild-type untagged YfeA was purified from the periplasm of an *E. coli* strain expressing the Yfe transporter *via* the previously characterized vector pYFE3, which contains the Yfe locus cloned from the *Y. pestis* genome (Bearden *et al.*, 1998 ▸; Bearden & Perry, 1999 ▸). Biochemical testing has shown that an *E. coli* strain harboring this vector is able to grow on EDDA-chelated medium that would otherwise abrogate growth; however, mutagenesis experiments on this vector indicate that all of the YfeABCDE components are required for growth, suggesting that pYFE3 recapitulates the full, functional Yfe transporter in *E. coli* (Bearden *et al.*, 1998 ▸; Bearden & Perry, 1999 ▸). Therefore, the YfeA produced from pYFE3 in *E. coli* is free to interact with its natural binding partners and produce a physiological metal signature. Under these conditions, native YfeA was purified by cell fractionation (Fig. 9 ▸ *a*) followed by anion-exchange and gel-filtration chromatography. Mass spectrometry confirmed the presence of the YfeA and YfeB components in whole cells, and only the YfeA component in the periplasm fraction (Figs. 9 ▸ *b* and 9 ▸ *c*). Further justification of our use of *E. coli* to produce relevant native YfeA protein was our finding that pre-pro YfeA was properly secreted to the periplasm with a cleaved signal peptide that was not present in the crystals of YfeA protein and was not detected by mass spectrometry. X-ray scattering data from ten crystals revealed that native YfeA crystallizes in the 42 × 52 × 113 Å crystal form. EDS spectra of native untagged YfeA crystals showed that the primary substrate at site 1 is zinc, with a slightly stronger signal from iron but surprisingly no signal from manganese (Fig. 6 ▸). Mysteriously, one out of 50 crystals gave a negligible EDS signature for all metals, indicating the stochastic nature of observing physiological apoprotein and suggesting the possibility of achieving apoprotein without the need for mutagenesis, partial denaturation or chelating agents. This alternative strategy instead requires the co-expression of binding partners and careful technique to extract protein while avoiding the reintroduction of the metal substrate that cluster A-1 SBPs can spontaneously bind. Efforts are currently under way to reproduce diffraction-quality crystals of this potentially apo YfeA.

### 3.7. Minor conformational changes in the flexible loops Trp96–Trp104, Val119–Pro140 and Tyr223–Gly233 and in helix 7 suggest that structural rearrangement occurs during metal loading and unloading   

**Figure 10:**
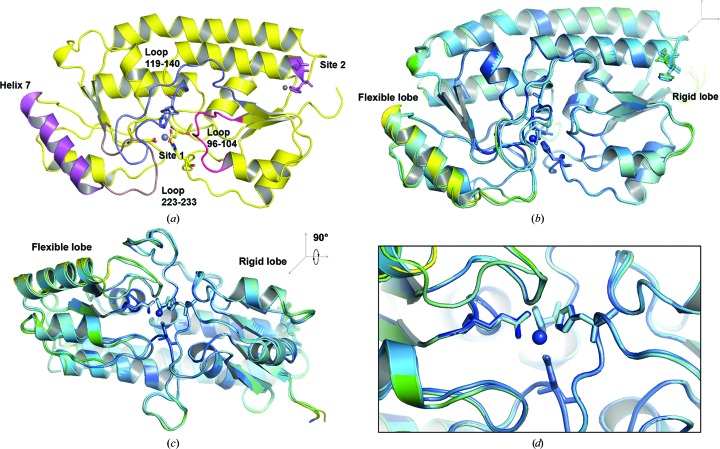
YfeA flexibility. The YfeA c-clamp is not entirely rigid. (*a*) Cartoon representation of YfeA illustrating the positions of the flexible helix and flexible loops relative to site 1 and site 2. (*b*) Superimposition of crystal forms colored by *B* factor shows subtle changes in the flexible loops and identifies a lone flexible helix in the carboxy-terminal lobe, annotated the ‘flexible lobe’. The absence of a flexible helix in the amino-terminal lobe suggests that this lobe is a ‘rigid lobe’. (*c*) Orthogonal view of (*b*) showing the proximity of the flexible helix and loop 223–233 relative to site 1. (*d*) Enlarged image of (*b*) at site 1 showing that ligands do not change position across crystal forms.

In this study, three crystal forms of YfeA were discovered, with minor differences between their respective structures (Fig. 10 ▸). Superimposition of the three crystal forms reveals minor conformational changes in the three flexible loops that flank site 1 (Figs. 10 ▸ *b* and 10 ▸ *c*), and *B*-factor analysis identifies a flexible helix, helix 7, that may participate in structural reorganization of the carboxy-terminal lobe during metal transfer (Fig. 10 ▸ *b*). The *B* factors for atoms in helix 7 reach a maximum in the 62 × 66 × 67 Å crystal form. The structural changes in the flexible loops all occur on the same face of the YfeA molecule, which is also the face that contains site 2 (Fig. 10 ▸ *a*). Crystal form 1 contains changes in loops 96–104 and 223–233, which deviate from their positions in crystal forms 2 and 3 (Figs. 10 ▸ *b* and 10 ▸ *c*). Loop 119–140 in crystal form 3 (the crystal form that arises from YfeA–EDTA co-crystallization) shows the greatest deviation from the other crystal forms. Although loop changes across crystal forms occur in close vicinity to site 1, there is a negligible perturbation of site 1 (Fig. 10 ▸ *d*). The concentration of these features on one face of the YfeA molecule but not the other may indicate the localization of a pipeline of allosteric changes extending from site 2 to site 1 (Fig. 10 ▸ *a*). Furthermore, these changes may reflect a greater degree of structural flexibility in cluster A-1 SBPs than may be anticipated.

## 4. Discussion   

YfeA is a polyspecific cluster A-1 SBP that is important for *Y. pestis* infection and transition-metal homeostasis (Bearden & Perry, 1999 ▸; Desrosiers *et al.*, 2010 ▸; Fetherston *et al.*, 2012 ▸; Perry *et al.*, 2012 ▸). YfeA contains a canonical metal-binding site that we refer to as site 1, with ligands that are evolutionarily conserved across SBPs from Gram-positive and Gram-negative genera (Fig. 5 ▸). EDS and anomalous X-ray scattering data confirm that site 1 binds zinc, iron and manganese in a manner that appears to be irreversible in the absence of physiological downstream binding partners for metal exchange. Site 1 appears to show a preference for zinc across all growth-condition experiments, including conditions where YfeA is produced in the context of its cognate transporter. It may be possible to achieve higher occupancies of manganese or iron by either adding more metal at the time of induction and harvesting cells sooner, or continuously supplementing with metal over the course of overexpression. However, such experiments would deviate from the physiological setting considerably given that the host and pathogen compete for limited metal nutrients.

Indeed, YfeA has been shown to bind zinc with high affinity but does not contribute to zinc accumulation in *Y. pestis* (Desrosiers *et al.*, 2010 ▸). To our knowledge, however, direct zinc-transport activity from the Yfe transporter, *i.e.* by ^65^Zn uptake, has not been measured. We cannot therefore distinguish between the possibility of potentially novel zinc-transport activity in YfeA and an artefactual interaction. We consider that our results highlight the phenomenon of the mismetallation of enzymes (Imlay, 2014 ▸) owing to the zinc occupancy being higher than those of manganese and iron in YfeA, which is a putative manganese and iron chaperone. Although oxidative stress is known to highly exacerbate mismetallation in mononuclear enzymes (Imlay, 2014 ▸), *E. coli* was not under oxidative stress to produce YfeA for this crystallographic study. Here, the cells were shaken heavily in an aerobic environment, and initially iron was not deficient, yet zinc was still the primary occupant at site 1. We are therefore unable to distinguish between mismetallization of YfeA with zinc or the more exciting possibility that YfeA, under some circumstances, may make some contribution to zinc uptake. Given that zinc is ubiquitous, under the conditions of YfeA overexpression the initial formation of apoprotein could lead to adventitious binding of zinc that is unrelated to any biological function. This could occur when the native metal substrate is relatively scarce or requires a chaperone for incorporation. For example, native azurin is a copper-binding periplasmic protein in *Pseudomonas aeruginosa* that is found to be zinc-bound after overexpression and purification from *E. coli* (Nar *et al.*, 1992 ▸). Zn-azurin is considered to be a contaminating byproduct from transport to the *E. coli* periplasm and not to be representative of the actual structure–function of *P. aeruginosa* Cu-azurin (Nar *et al.*, 1992 ▸). Another example is PsaA, which is a manganese-binding SBP in *Streptococcus pneumoniae* that also binds zinc, although Mn-PsaA and Zn-PsaA are nearly structurally identical (McDevitt *et al.*, 2011 ▸; Couñago *et al.*, 2014 ▸). Biophysical experiments on PsaA revealed that Zn-PsaA is more thermally stable than Mn-PsaA, and that the affinity of PsaA for zinc is nearly two orders of magnitude greater than the affinity of PsaA for manganese (McDevitt *et al.*, 2011 ▸; Couñago *et al.*, 2014 ▸). However, intracellular metal-accumulation measurements indicate that PsaA does not transport zinc (McDevitt *et al.*, 2011 ▸; Couñago *et al.*, 2014 ▸). Intriguingly, PsaA has also been shown to bind and transport cadmium, in spite of cadmium not being a physiological substrate (Begg *et al.*, 2015 ▸). An alternative explanation for YfeA being zinc-bound might be that potential apo YfeA could quickly become metal-bound during cell lysis from leaked cytoplasmic contents prior to purification, further complicating the interpretation of bound zinc.

The metal-binding profile for YfeA site 1 can vary according to environmental conditions, as the relative abundance of any bound substrate can change. Although the profile of the bound substrate can change, EDTA cannot remove metal cargo from site 1, even after including EDTA in the purification buffers, overnight dialysis and co-crystallization. This suggests that the only way to remove the metal from site 1 is by interaction with downstream YfeA-binding partners and a major conformational change or degradation mechanism. The observation that even extensive treatment with EDTA cannot remove the metal from YfeA site 1 makes YfeA distinct from other SBPs that have been produced in an apo state, such as ZinT from *Salmonella enterica* (Ilari *et al.*, 2014 ▸). In the literature, apo SBPs have been generated through mutagenesis, partial denaturation and/or EDTA treatment. Therefore, future studies to produce apo YfeA might include these strategies as well as overexpression of YfeA in the context of its endogenous binding partners.

Metal-coordination geometry alone cannot unequivocally exclude the possibility of binding one metal over another; however, tetrahedral geometries are known for zinc, iron and manganese, although they are not as common as other geometries for iron and manganese (Dokmanić *et al.*, 2008 ▸; Tus *et al.*, 2012 ▸). Rubredoxin is an example of an iron-containing protein with a tetrahedral site (Eaton & Lovenberg, 1970 ▸). PsaA is an example of a manganese-containing protein with a tetrahedral site, with metal-binding ligands that are accessible in the apo state and buried in the holo state (Couñago *et al.*, 2014 ▸). In tetracoordinate geometry, zinc, manganese and iron prefer a tetrahedral or near-tetrahedral geometry in which all four ligands are orthogonal to each other and provide a stronger interaction with the metal. Given that tetrahedral geometry is less common in iron and manganese, the geometry of YfeA site 1 may influence competitive metal-binding affinities such that when the metal-binding geometry is a poor fit interaction with this metal is weaker. This may also explain differences in site 1 metal occupancies as well as the *K*_d_ for YfeA–Zn and YfeA–Mn interactions (Desrosiers *et al.*, 2010 ▸), although the ITC data are confounded by the discovery of site 2 and the ancillary sites. To specifically measure the canonical site affinity for zinc, manganese or iron, mutagenesis of all of the other metal-binding sites in this report would be required for comparative analysis, highlighting the need for rigorous structural investigation of any SBP and the elucidation of additional metal-binding sites.

From a coordination-geometry perspective, the hierarchy of metal binding at site 1 is expected to be Zn >>> Fe > Mn based on the frequency of tetrahedral geometry observed in protein–metal binding in the PDB (Dokmanić *et al.*, 2008 ▸; Tus *et al.*, 2012 ▸); however, this is a relative binding preference and not an absolute preference, as the X-ray fluorescence data suggest that the site 1 metal preference is Zn >>> Mn > Fe. Therefore, specificity for zinc, manganese or iron at site 1 must not be determined by coordination geometry but rather by some other property. In *E. coli*, the intracellular manganese concentration is very low relative to those of other metals during normal growth conditions (Anjem *et al.*, 2009 ▸). However, manganese seems to function as a metabolic oxidant-resistant substitute for iron in metallating mononuclear enzymes under conditions of iron starvation and oxidative stress (Anjem *et al.*, 2009 ▸). We believe that mangan­ese serves a similar role in *Y. pestis*, and that the Yfe transporter has a physiological preference for manganese that is greater than that for iron based on the negligible manganese signal relative to the iron signal in the EDS spectra of purified native YfeA that was overexpressed in the context of YfeBCDE. A negligible manganese signal suggests that manganese transport may be more rapid than iron transport through YfeBCDE. The Yfe transporter may actually serve as a conduit to transition between iron-based metabolism and manganese-based metabolism. We speculate that this key role is the reason that YfeA is important for virulence and is upregulated significantly more than other SBPs during *Y. pestis* infection. At this time, the role of zinc is unclear.

Manganese co-crystallization experiments revealed site 2, which is a surface metal-binding site that prefers to spontaneously bind zinc but can spontaneously bind manganese as well. Zinc can be titrated in a co-crystallization experiment to increase its occupancy at site 2 but, curiously, manganese is required for loading. The reason that manganese is required is unknown and cannot be determined crystallographically. Back-soaking experiments indicate that site 2 metal loading is irreversible and only occurs when YfeA is aqueous prior to crystallization. We speculate that site 2 may play a functional role in communication with the Yfe transporter. Soaking YfeA crystals with zinc revealed nine ancillary metal-binding sites, half of which only load when site 2 is occupied and the other half of which only load when site 2 is apo. It is curious that the ancillary sites that load with site 2 mostly occur in the immediate proximity of site 2 rather than at distal sites on the molecule. Site 2 and the ancillary sites demonstrate that YfeA can continue to take up free metal from solution after site 1 is loaded.

Site 2 binds manganese and zinc with tetrahedral or near-tetrahedral geometry by additional coordination from two water molecules. Cations bound to site 2 in the YfeA crystal may readily dissociate in solution, particularly because the free metal concentration in the periplasm is low. The combination of site 2 being a surface site and the observation of water molecules coordinating the metal suggests that bound cations are easily accessible and readily interchangeable, further supporting a role in communication with the Yfe transporter. Histidine is the most common recurring ligand that chelates zinc, iron and manganese in the PDB (Dokmanić *et al.*, 2008 ▸; Tus *et al.*, 2012 ▸). The ligands and geometries of sites 1 and 2 may have electronic significance, such as tuning the redox properties of each site and mitigating metal transfer. At site 2, we believe that histidine may be the more crucial ligand and that glutamate may help to tune histidine–metal binding. Future directions include mutagenesis studies of site 2 and functional investigation of site 2 in *Y. pestis* YfeA.

Metal-soaking experiments were performed using 10 m*M* ZnCl_2_, which is well above the physiological zinc concentration; thus, occupancy during normal growth conditions may not occur. Instead, this function may be important under high (but not toxic) zinc levels when iron or manganese levels are low (Desrosiers *et al.*, 2010 ▸). During infection, an antibacterial strategy used by phagocytes is respiratory burst, or the rapid release of highly reactive chemicals designed to be toxic in nature and to catalyze the formation of additional toxic chemicals (Slauch, 2011 ▸). The ancillary sites may also allow YfeA to loosely bind Zn atoms and act as a zinc sink when zinc is in toxic excess, such as during respiratory burst. All YfeA ancillary metal-binding sites are composed of single ligands that are likely to be weak chelators; however, given that they are on the surface and have been shown to interact with zinc, they may play a role in this speculative activity. Metals in these ancillary sites can be exchanged *via* back-soaking, indicating that binding to these sites is reversible. It is worth noting that another *Y. pestis* SBP has been shown to contain multiple ancillary single-ligand sites, although the relevance of these sites was proposed to be artefactual (Shouldice *et al.*, 2005 ▸).

A topic of debate in cluster A-1 SBPs is the precise mechanism of metal loading and unloading at the canonical metal-binding site. By varying the growth and crystallization conditions, we observed three subtle conformational changes in the YfeA flexible loops that flank site 1 and a flexible helix in the carboxy-terminal lobe. Intriguingly, a flexible loop in ZnuA that is in a similar position to the YfeA flexible loop 119–140 has been proposed to influence zinc transfer (Wei *et al.*, 2007 ▸). We speculate that a major structural rearrangement to the c-clamp must be required to unload metal from site 1, and in the case of YfeA this rearrangement will occur through helix 7 in the flexible lobe. We anticipate the structurally rearranged state to be unstable for crystallography, and that crystallography may actually select for the more stable, intact c-clamp. Although there may be no metal bound, the observed apo protein may simply be protein that has structurally reverted to an intact c-clamp. Structural comparisons of many apo and metal-bound cluster A-1 SBPs indicate that there is very little movement in the α/β domains between the states (Andrews *et al.*, 2003 ▸; Couñago *et al.*, 2012 ▸). Should a structural rearrangement occur during metal transfer that forces YfeA or other cluster A-1 SBPs into an unstable state that is not an intact c-clamp, then this might suggest that the true apo state of cluster A-1 SBPs may not yet have been observed.

## Supplementary Material

PDB reference: YfeA, 5uxs

PDB reference: YfeA + EDTA, 5uxu

PDB reference: YfeA, site 2 (λZn), 5uyg

PDB reference: YfeA, site 2 (λMn), 5uyh

PDB reference: YfeA, apo site 2, ancillary sites, 5uyv

PDB reference: YfeA, bound site 2, ancillary sites, 5uyw

PDB reference: YfeA, M9–Fe (λZn), 5uy0

PDB reference: YfeA, M9–Fe (λFe), 5uy4

PDB reference: YfeA, M9–Fe (λMn), 5uy5

PDB reference: YfeA, M9–Mn (λZn), 5uya

PDB reference: YfeA, M9–Mn (λFe), 5uyb

PDB reference: YfeA, M9–Mn (λMn), 5uyc

PDB reference: YfeA, M9–Zn (λZn), 5uyd

PDB reference: YfeA, M9–Zn (λFe), 5uye

PDB reference: YfeA, M9–Zn (λMn), 5uyf

## Figures and Tables

**Table 1:** Data-collection and refinement statistics Values in parentheses are for the highest resolution shell.

	YfeA (λZn)	EDTA (λZn)	Site 2 (λZn)	Site 2 (λMn)	Apo site 2, ancillary sites (λZn)	Bound site 2, ancillary sites (λZn)
PDB code	5uxs	5uxu	5uyg	5uyh	5uyv	5uyw
Data collection						
Beamline	SER-CAT, APS	GM-CA, APS	SER-CAT, APS	SER-CAT, APS	GM-CA, APS	SER-CAT, APS
Wavelength (Å)	1.28281	1.28229	1.28281	1.89288	1.27932	1.28281
Space group	*P*2_1_2_1_2_1_	*P*2_1_2_1_2_1_	*P*2_1_2_1_2_1_	*P*2_1_2_1_2_1_	*P*2_1_2_1_2_1_	*P*2_1_2_1_2_1_
*a*, *b*, *c* (Å)	41.74, 52.12, 113.07	55.32, 66.88, 81.69	62.36, 66.04, 66.83	62.39, 66.10, 66.83	41.52, 51.96, 112.75	62.28, 66.36, 67.09
α = β = γ (°)	90	90	90	90	90	90
*V*_M_ † (Å^3^ Da^−1^)	1.98	2.43	2.21	2.21	1.95	2.23
Solvent content (%)	37.77	49.36	44.38	44.46	37.07	44.80
Resolution (Å)	50.00–1.42 (1.44–1.42)	50.00–1.84 (1.87–1.84)	50.00–1.86 (1.89–1.86)	50.00–1.99 (2.02–1.99)	50.00–1.69 (1.72–1.69)	50.00–1.95 (1.98–1.95)
Unique reflections	47252 (2340)	26517 (1199)	23851 (1183)	19483 (940)	27201 (1217)	20672 (998)
Completeness (%)	99.7 (99.6)	98.0 (90.6)	99.8 (99.9)	99.3 (96.9)	96.8 (88.4)	99.6 (98.4)
Multiplicity	4.6 (4.5)	3.3 (2.6)	4.8 (4.8)	4.6 (4.0)	3.2 (2.7)	4.8 (4.8)
CC_1/2_	98.1 (85.4)	99.5 (92.4)	98.6 (90.3)	99.5 (83.3)	94.8 (75.2)	99.6 (97.4)
*R*_merge_ (%)	9.8 (40.1)	6.6 (17.1)	7.1 (33.5)	6.4 (40.3)	8.2 (25.9)	7.1 (42.1)
*R*_meas_ (%)	11.1 (45.6)	7.9 (21.1)	8.0 (42.6)	7.2 (46.2)	9.7 (32.1)	8.0 (47.1)
*R*_p.i.m._ (%)	5.1 (21.3)	4.2 (12.2)	3.5 (18.8)	3.2 (22.2)	5.2 (18.6)	3.6 (20.7)
Mean *I*/σ(*I*)	52.8 (2.2)	48.9 (0.5)	46.6 (0.4)	53.6 (0.4)	43.7 (4.1)	44.9 (0.7)
Refinement						
Resolution (Å)	33.58–1.42 (1.44–1.42)	40.86–1.84 (1.86–1.84)	46.99–1.86 (1.88–1.86)	47.01–1.99 (2.02–1.99)	38.97–1.69 (1.71–1.69)	31.14–1.95 (1.98–1.95)
No. of non-anomalous reflections	47185	26477	23804	19437	27168	20628
Completeness (%)	99.4 (97.0)	94.5 (81.1)	99.9 (99.1)	99.2 (96.4)	90.8 (77.6)	99.5 (94.7)
*R*_work_ (%)	17.88 (25.64)	17.03 (23.63)	18.92 (24.74)	18.56 (31.23)	17.31 (27.62)	18.66 (26.28)
*R*_free_ ‡ (%)	19.03 (29.52)	20.30 (23.16)	21.80 (29.36)	23.53 (30.36)	20.61 (34.63)	22.39 (32.42)
Wilson *B* factor (Å^2^)	15.596	19.045	28.509	36.649	17.145	27.528
Average *B* factor (Å^2^)	21.99	22.57	35.44	41.59	19.86	35.52
No. of protein atoms	2153	2161	2140	2159	2154	2140
Solvent atoms	365 H_2_O, 1 Zn	404 H_2_O, 1 Zn	250 H_2_O, 2 Zn	179 H_2_O, 2 Mn	363 H_2_O, 6 Zn	188 H_2_O, 6 Zn
No. of molecules in ASU§	1	1	1	1	1	1
R.m.s.d.¶, bond lengths (Å)	0.006	0.007	0.008	0.008	0.007	0.008
R.m.s.d., bond angles (°)	0.76	0.875	0.887	0.894	0.841	0.907
Ramachandran plot						
Favored (%)	97.06	97.8	96.67	97.78	96.69	95.56
Allowed (%)	2.21	1.47	2.96	2.22	2.57	3.7
Outliers (%)	0.74	0.73	0.37	0	0.74	0.74
Clashscore	2.79	3.24	7.47	5.37	4.87	7.46
*MolProbity* score	1.23	1.16	1.62	1.34	1.46	1.72

† Matthews coefficient.

‡ The test set uses ∼5% of the data.

§ Asymmetric unit.

¶ Root-mean-square deviation.

**Table 2:** Data-collection and refinement statistics Values in parentheses are for highest resolution shell.

	M9–Fe (λZn)	M9–Fe (λFe)	M9–Fe (λMn)	M9–Mn (λZn)	M9–Mn (λFe)	M9–Mn (λMn)	M9–Zn (λZn)	M9–Zn (λFe)	M9–Zn (λMn)
PDB code	5uy0	5uy4	5uy5	5uya	5uyb	5uyc	5uyd	5uye	5uyf
Data collection									
Beamline	CMCF, CLS	CMCF, CLS	CMCF, CLS	CMCF, CLS	CMCF, CLS	CMCF, CLS	CMCF, CLS	CMCF, CLS	CMCF, CLS
Wavelength (A)	1.28215	1.74013	1.89174	1.28215	1.74013	1.89289	1.28215	1.74013	1.89289
Space group	*P*2_1_2_1_2_1_	*P*2_1_2_1_2_1_	*P*2_1_2_1_2_1_	*P*2_1_2_1_2_1_	*P*2_1_2_1_2_1_	*P*2_1_2_1_2_1_	*P*2_1_2_1_2_1_	*P*2_1_2_1_2_1_	*P*2_1_2_1_2_1_
*a*, *b*, *c* (Å)	61.93, 66.47, 67.25	61.93, 66.37, 67.15	61.97, 66.49, 67.21	41.79, 52.13, 113.01	41.75, 52.19, 112.75	41.73, 52.18, 112.78	41.79, 52.17, 112.80	41.81, 52.17, 112.77	41.86, 52.19, 112.77
α = β = γ (°)	90	90	90	90	90	90	90	90	90
*V*_M_ † (Å^3^ Da^−1^)	2.22	2.22	2.23	1.98	1.97	1.97	1.98	1.98	1.98
Solvent content (%)	44.71	44.54	44.73	37.83	37.69	37.67	37.76	37.77	37.87
Resolution (Å)	50.00–1.71 (1.74–1.71)	50.00–1.92 (1.95–1.92)	50.00–2.15 (2.19–2.15)	50.00–1.72 (1.75–1.72)	50.00–1.95 (1.98–1.95)	50.00–1.96 (1.99–1.96)	50.00–1.93 (1.96–1.93)	50.00–2.09 (2.13–2.09)	50.00–2.06 (2.10–2.06)
Unique reflections	30412 (1478)	21484 (1046)	15410 (654)	26994 (1320)	18617 (921)	18167 (784)	19305 (937)	15274 (713)	15986 (800)
Completeness (%)	98.9 (97.7)	98.0 (96.1)	97.5 (85.4)	99.8 (98.7)	99.7 (99.9)	98.9 (88.3)	99.9 (99.9)	99.9 (99.9)	99.9 (99.9)
Multiplicity	8.1 (8.1)	7.9 (7.8)	7.8 (6.5)	7.4 (7.3)	7.6 (7.5)	7.3 (4.3)	7.2 (7.2)	7.3 (7.4)	7.2 (6.9)
CC_1/2_	99.7 (93.8)	99.7 (93.4)	99.7 (95.4)	99.5 (96.5)	99.5 (94.1)	99.6 (74.7)	99.5 (93.7)	99.5 (94.1)	99.5 (88.3)
*R*_merge_ (%)	8.5 (40.4)	6.3 (41.4)	6.5 (24.0)	9.6 (40.4)	9.2 (40.2)	9.4 (37.9)	9.6 (42.6)	9.4 (41.8)	9.3 (41.1)
*R*_meas_ (%)	9.0 (43.1)	6.8 (44.3)	7.0 (26.1)	10.3 (43.3)	9.8 (43.3)	10.1 (43.5)	10.3 (45.9)	10.2 (44.9)	10.0 (44.6)
*R*_p.i.m._ (%)	3.2 (15.0)	2.4 (15.5)	2.5 (9.9)	3.7 (15.4)	3.5 (15.8)	3.6 (20.8)	3.8 (16.9)	3.7 (16.4)	3.7 (17.1)
Mean *I*/σ(*I*)	56.0 (1.4)	56.0 (3.1)	67.4 (14.0)	59.1 (7.9)	52.7 (5.2)	73.2 (13.3)	54.8 (6.4)	51.3 (6.0)	67.3 (13.5)
Refinement									
Resolution (Å)	30.97–1.71 (1.73–1.71)	47.22–1.92 (1.94–1.92)	37.59–2.14 (2.18–2.14)	38.32–1.72 (1.74–1.72)	39.16–1.95 (1.98–1.95)	38.29–1.96 (1.99–1.96)	39.19–1.92 (1.95–1.92)	39.21–2.09 (2.12–2.09)	38.30–2.06 (2.09–2.06)
No. of non-anomalous reflections	30366	21444	15376	26936	21444	18116	19252	15227	15934
Completeness (%)	98.9 (96.5)	97.8 (89.4)	97.6 (82.8)	99.5 (93.9)	99.5 (96.6)	98.6 (82.8)	99.7 (91.9)	99.9 (97.1)	99.6 (92.5)
*R*_work_ (%)	17.85 (23.69)	17.28 (29.27)	16.34 (18.19)	16.45 (18.40)	16.51 (23.26)	16.83 (29.84)	16.19 (22.62)	16.18 (19.11)	16.24 (22.29)
*R*_free_ ‡ (%)	21.24 (23.13)	20.14 (32.99)	20.40 (23.83)	19.22 (21.57)	20.17 (30.77)	20.29 (33.45)	20.11 (28.45)	20.68 (26.04)	20.04 (23.20)
Wilson *B* factor (Å^2^)	27.603	27.43	33.109	20.66	22.293	26.003	22.727	26.184	34.315
Average *B* factor (Å^2^)	33.14	32.52	37.75	24.92	26.66	29.43	25.87	29	37.06
No. of protein atoms	2133	2133	2133	2153	2133	2153	2153	2153	2153
Solvent atoms	285 H_2_O, 1 Zn	245 H_2_O, 1 Fe	180 H_2_O, 1 Mn	293 H_2_O, 1 Zn	245 H_2_O, 1 Fe	229 H_2_O, 1 Mn	284 H_2_O, 1 Zn	211 H_2_O, 1 Fe	181 H_2_O, 1 Mn
No. of molecules in ASU§	1	1	1	1	1	1	1	1	1
R.m.s.d.¶, bond lengths (Å)	0.007	0.007	0.008	0.006	0.006	0.007	0.007	0.007	0.006
R.m.s.d.¶, bond angles (°)	0.808	0.805	0.832	0.775	0.764	0.799	0.774	0.804	0.766
Ramachandran plot									
Favored (%)	98.14	98.14	98.51	96.69	98.14	96.69	96.32	96.69	97.06
Allowed (%)	1.86	1.86	1.49	2.21	1.86	2.57	2.94	2.57	2.21
Outliers (%)	0	0	0	1.1	0	0.74	0.74	0.74	0.74
Clashscore	5.39	4.45	4.92	4.18	4.41	5.11	3.71	4.41	2.55
*MolProbity* score	1.29	1.22	1.26	1.41	1.39	1.48	1.41	1.43	1.21

† Matthews coefficient.

‡ The test set uses ∼5% of the data.

§ Asymmetric unit.

¶ Root-mean-square deviation.
